# Glucocorticoids associate with cardiometabolic risk factors in black South Africans

**DOI:** 10.1530/EC-21-0195

**Published:** 2021-07-14

**Authors:** Siphiwe N Dlamini, Zané Lombard, Lisa K Micklesfield, Nigel Crowther, Shane A Norris, Tracy Snyman, Andrew A Crawford, Brian R Walker, Julia H Goedecke

**Affiliations:** 1 SAMRC/Wits Developmental Pathways for Health Research Unit (DPHRU), School of Clinical Medicine, Faculty of Health Sciences, University of the Witwatersrand, Johannesburg, South Africa; 2 Non-communicable Diseases Research Unit, South African Medical Research Council, Cape Town, South Africa; 3 Division of Human Genetics, National Health Laboratory Service, and School of Pathology, Faculty of Health Sciences, University of the Witwatersrand, Johannesburg, South Africa; 4 Department of Chemical Pathology, National Health Laboratory Service and Faculty of Health Sciences, University of the Witwatersrand, Johannesburg, South Africa; 5 Population Health Sciences, Bristol Medical School, University of Bristol, Bristol, UK; 6 BHF Centre for Cardiovascular Science, University of Edinburgh, Edinburgh, UK; 7 Institute of Genetic Medicine to Translational & Clinical Research Institute, Newcastle University, Newcastle upon Tyne, UK

**Keywords:** glucocorticoids, metabolic syndrome, cardiometabolic risk factors, cortisol, corticosterone

## Abstract

Circulating glucocorticoids are associated with metabolic syndrome and related cardiometabolic risk factors in non-Africans. This study investigated these associations in Africans, whose metabolic phenotype reportedly differs from Europeans. Adiposity, blood pressure, glycaemia, insulin resistance, and lipid profile, were measured in 316 African men and 788 African women living in Soweto, Johannesburg. The 2009 harmonized criteria were used to define metabolic syndrome. Serum glucocorticoids were measured using liquid chromatography-mass spectrometry. Cortisol was associated with greater odds presenting with metabolic syndrome (odds ratio (95% CI) =1.50 (1.04, 2.17) and higher systolic (beta coefficient, β (95% CI) =0.04 (0.01, 0.08)) and diastolic (0.05 (0.02, 0.09)) blood pressure, but higher HDL (0.10 (0.02, 0.19)) and lower LDL (−0.14 (−0.24, −0.03)) cholesterol concentrations, in the combined sample of men and women. In contrast, corticosterone was only associated with higher insulin sensitivity (Matsuda index; 0.22 (0.03, 0.41)), but this was not independent of BMI. Sex-specific associations were observed, such that both cortisol and corticosterone were associated with higher fasting glucose (standardized β (95% CI): 0.24 (0.12, 0.36) for cortisol and 0.12 (0.01, 0.23) for corticosterone) and HbA1c (0.13 (0.01, 0.25) for cortisol and 0.12 (0.01, 0.24) for corticosterone) in men only, but lower HbA1c (0.10 (−0.20, −0.01) for cortisol and −0.09 (−0.18, −0.03) for corticosterone) in women only. Our study reports for the first time that associations between circulating glucocorticoid concentrations and key cardiometabolic risk factors exhibit both glucocorticoid- and sex-specificity in Africans.

## Introduction

Elevated waist circumference, blood pressure, fasting glucose, triglycerides, and reduced high-density lipoprotein (HDL) cholesterol often cluster together as components of metabolic syndrome (1). Chronic exposure to excess cortisol leads to Cushing’s syndrome, which exhibits metabolic features similar to metabolic syndrome (2). Accordingly, previous cross-sectional studies suggest that circulating cortisol concentrations are associated with metabolic syndrome, its components, and related cardiometabolic risk factors, including measures of obesity, insulin resistance, and glucose intolerance (3, 4, 5, 6, 7).

Cortisol may not be the only glucocorticoid involved in metabolic syndrome as humans also produce corticosterone. As cortisol production is almost ten times higher than corticosterone (8), previous studies have only focused on cortisol and assumed that these two glucocorticoids have identical functions (9). However, recent evidence suggests that cortisol and corticosterone may have distinct roles in human health and disease (10).

Notably, most of the reported cross-sectional studies that have explored the associations between cortisol and cardiometabolic risk factors were conducted in non-Africans (3, 4, 5, 6). Africans have a different metabolic phenotype to that of their European counterparts (11, 12). Some studies have shown that in urban settings Africans have a higher prevalence of obesity and insulin resistance, yet have less visceral adipose tissue (VAT) than Europeans (13, 14, 15). Although the mechanisms underlying these ethnic differences are not yet known, a recent study suggested that glucocorticoids may be involved (16). Goedecke *et al.* (16) reported that black South African women demonstrated lower gene expression of the glucocorticoid receptor (GR) in s.c. adipose tissue (SAT) than women of European ancestry. The lower GR expression in these African women was associated with less VAT, higher SAT inflammation, and whole-body lower insulin sensitivity. Furthermore, some studies have shown that circulating cortisol concentrations are lower in African women compared to women of European ancestry (17, 18). In support of these ethnic differences, a study that included both African men and women suggested more evidence of hypothalamic–pituitary–adrenal (HPA) axis dysregulation in African males compared to their European ancestry counterparts, and the HPA axis dysregulation was associated with an increased risk of cardiovascular disease (7). Based on the above observations, we hypothesized that the often-observed ethnic differences in body fat distribution, inflammatory profile, insulin sensitivity, and circulating cortisol concentrations are partly explained by tissue-specific differences of glucocorticoid metabolism within SAT.

However, as studies of cortisol and metabolic syndrome in Africans are scarce and none have included corticosterone, it is still uncertain whether circulating glucocorticoids associate with metabolic syndrome and related cardiometabolic risk factors in Africans (19, 20, 21). Therefore, the aim of this study was to investigate the associations between fasting serum corticosterone and cortisol concentrations and metabolic syndrome and related cardiometabolic risk factors in black South African men and women.

## Materials and methods

### Study population and sampling procedure

The study sample consisted of adult male and female participants recruited from various studies at the SAMRC/Wits Developmental Pathways for Health Research Unit, based at the Chris Hani Baragwanath Academic Hospital in Soweto, Johannesburg. Study sample 1 (ethical clearance M090620) included women recruited as part of the Africa Wits-INDEPTH partnership for Genomic Research (AWI-Gen) study (22). These women (*n* = 1007) who were randomly recruited between 2011 and 2013 were the caregivers of the birth to twenty plus (Bt20+) cohort. Bt20+ is an ongoing longitudinal study, and its profile has been described elsewhere (23). The majority of the caregivers of the Bt20+ participants are South African men and women of African ancestry who were self-identified as South-Eastern Bantu language speakers, and only this ethnic group was included in the present study. Details of data collection protocols for the AWI-Gen study have been described elsewhere (24). Study sample 2 included participants (501 men and 529 women) recruited as part of a longitudinal study designed to identify the determinants of type 2 diabetes risk in middle-aged South African men and women (ethical clearance M160604). These participants were a sub-sample of the AWI-Gen study from whom follow-up data collected between January 2017 and August 2018 were used and included no participants from study sample 1.

**Figure 1:**
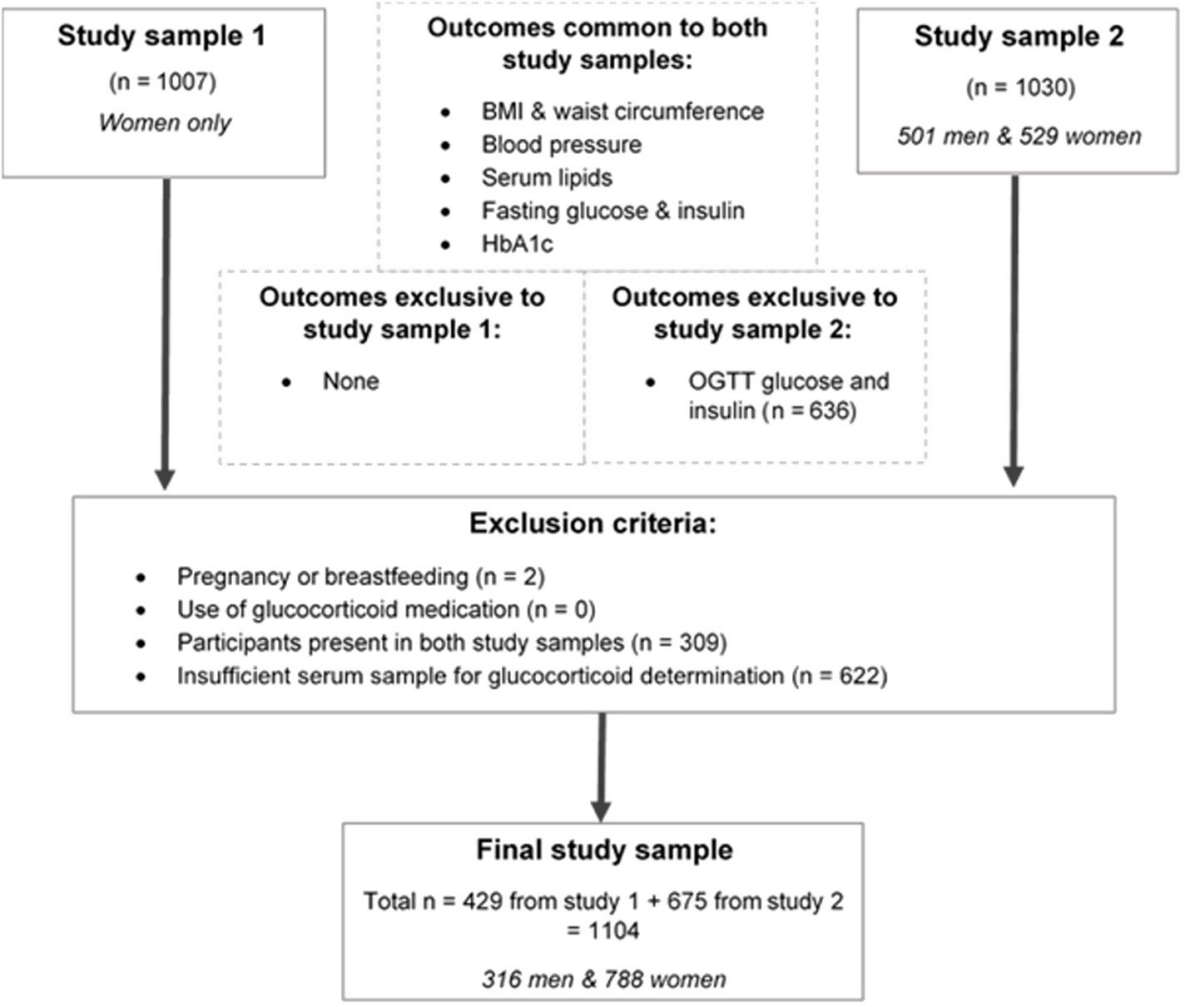
Selection of the study sample.

The selection of the participants for the present study was primarily based on the availability of serum samples for glucocorticoid determination. Participants who were pregnant or using corticosteroid medication were excluded from the study (Fig. 1). A total of 1104 participants were selected for the present study, with the selection process described in Fig. 1. Consent was obtained from all study participants following a full explanation of the purpose and nature of all procedures used. Further ethical clearance for all analyses conducted as part of the present study was also obtained (ethical clearance no: M160225) from the University of the Witwatersrand Human Research Ethics Committee (Medical).

### Participant testing procedures

Participants were asked to remove heavy clothes and shoes prior to weight and height measurements. Weight was measured using a calibrated digital scale (Dismed, Halfway House, SA) to the nearest 0.1 kg. Standing height was measured using a fixed-wall stadiometer (Holtain, Crymych, UK) to the nearest 0.1 cm. BMI was calculated as weight (kilograms) divided by height squared (metre square). Waist circumference was determined at the level of the umbilicus when the participants were standing and measured using a soft measuring tape to the nearest 0.5 cm. Systolic and diastolic blood pressure were measured on the left arm using a digital blood pressure reader (Omron M6, Kyoto, Japan) and appropriate cuffs. After the participant had been seated for at least 5 min, three blood pressure readings were taken at 2-min intervals, and the average of the second and third readings was recorded and used in the analyses. Participants who self-reported as smoking any form of tobacco and/or drinking any form of alcohol were classified as current smokers or drinkers, respectively. Participants were asked to bring their chronic medication to the interview sessions to confirm their disease status.

### Blood sampling and biochemical analyses

Standard venepuncture techniques were used to collect blood samples after 10–12 h of fasting for the subsequent determination of serum lipids, HbA1c, serum insulin, plasma glucose, and serum glucocorticoid concentrations. Blood sampling time for the fasting samples was recorded and ranged from 07:00 to 11:59 h. To convert the blood sampling time into a more continuous variable, minutes from the earliest blood sample (07:00 h) were calculated. For example, a blood sample collected at 08:00 h was assigned a time value of 60 min. A standard oral glucose tolerance test (OGTT) was conducted on a sub-sample of participants (Study 2 in Fig. 1, *n* = 636). During the OGTT, the participants ingested 75 g anhydrous glucose dissolved in 250 mL water. Blood samples were then collected at 30-min intervals for 120 min for the subsequent determination of the Matsuda index (OGTT-derived insulin sensitivity) (http://mmatsuda.diabetes-smc.jp/english.html) and 2-h OGTT glucose concentrations.

Serum lipid concentrations, including triglycerides and total- and HDL-cholesterol, were measured using prescribed enzymatic methods with the RX Daytona Chemistry Analyser (Randox, Crumlim, UK). The inter- and intra-assay coefficients of variation (CVs) for the serum lipids were 1.4 and 1.5% for triglycerides, respectively, both 1.3% for total cholesterol and 1.7 and 1.8% for HDL cholesterol, respectively. Serum low-density lipoprotein (LDL)-cholesterol concentrations were calculated using the Friedewald equation (25). The HbA1c concentrations were measured in whole blood samples using the D-10 haemoglobin testing system (BioRad), and the inter- and intra-assay CVs were both 1.8%. Plasma glucose concentrations were measured using the RX Daytona Chemistry Analyser (Randox), and the Advia 1800 Chemistry Analyser (Siemens Healthcare Diagnostics) was used to measure serum insulin concentrations. The inter- and intra-assay CVs were 3.3 and 1.3% for glucose, respectively and 2.9 and 2.7% for insulin, respectively. The HOMA2-IR calculator version 2.2.3 was used to estimate insulin resistance levels (https://www.dtu.ox.ac.uk/homacalculator) from fasting serum insulin and plasma glucose concentrations.

### Definition of metabolic syndrome

The 2009 harmonized guidelines were used for the diagnosis of metabolic syndrome (1). The harmonized diagnosis requires the presence of three or more of the following criteria: elevated waist circumference (≥94 cm in men, ≥80 cm in women), elevated triglyceride (≥1.7 mmol/L), low HDL cholesterol (<1.0 mmol/L in males, <1.3 mmol/L in females), elevated blood pressure (systolic ≥ 130 and/or diastolic ≥ 85 mmHg), and elevated fasting glucose (≥5.6 mmol/L). The use of prescribed medications for elevated blood pressure and diabetes were also considered as relevant indicators of the presence of elevated blood pressure and fasting blood glucose concentrations, respectively.

### Steroid extraction and glucocorticoid measurements

#### Extraction of steroids from serum

All calibrators, internal standards (labelled with stable isotope deuterium; corticoseterone-d8 and cortisol-d4), and controls were purchased from Chromsystems (Grafelfing, Germany). A liquid–liquid extraction technique (Agilent technologies) was used to extract all steroids. Briefly, 50 μL of the internal standard was added to 500 μL of serum. Three millilitres of methyl tert-butyl ether (MTBE) were added to each sample and vortexed at maximum speed of 3 × 30 s, with 30 s rests in between. The sample was then centrifuged at 1372 ***g*** for 10 min. The supernatant was transferred into a clean glass tube and dried down using technical grade nitrogen (Afrox, Johannesburg, SA) at room temperature. The extracted steroids were reconstituted in 100 μL of 50% methanol: water and then transferred into limited volume analytical vials (Kinesis inc., Vernon Hills, USA) for analyses on the ultra-higher pressure liquid chromatography (UPLC) mass spectrometry (MS).

#### Glucocorticoid measurement in the UPLC-MS

Ten microlitres of each extracted sample were injected onto a Kinetex F5 2.6 μm 50 × 2.1 mm column (Phenomenex, California, USA) utilizing an Acquity UPLC that was connected to a Xevo-TQS MS (Waters Corporation, Milford, USA). The UPLC-MS method was optimized and validated to quantitate both corticosterone and cortisol. A flow rate of 0.6 mL/min set on a gradient profile was used to elute the compounds. A binary flow mobile phase was used and consisted of mobile phase A: 5 mM ammonium formate and 0.1% formic acid in deionized water and mobile phase B: 1 mM ammonium formate and 0.1% formic acid in acetonitrile to elute off each of the glucocorticoids over a total run time of 5.50 min. The gradient programme was set as follows: 5% B for the first 0.95 min; 30% B for 0.5 min; 70% B for 0.50 min; this was ramped to 90% B at 1.95 min and maintained for 2.55 min with a final ramp to 95% B for 0.50 min to wash the column. The conditions were then changed back to initial starting conditions (5% B) at 4 min to equilibrate the column. The retention time for corticosterone and corticosterone-d8 was 3.23 and 3.25 min, respectively, whereas the retention time for cortisol and cortisol-d4 was 3.35 and 3.36 min, respectively.

Electrospray ionization in positive mode was used in the MS. Capillary voltage was 3 kV with cone and collision energies optimized to each compound analysed. Source temperature and desolvation temperature were set at 150° and 350°C, respectively. Cone gas was set at 550 L/h, and desolvation gas was set at 150 L/h. The analytes were identified with multiple reaction monitoring identified for each individual compound. Corticosterone had a transition of 347>121 as the quanitifier and 347>329 for the qualifier. Cortisol utilized the transition of 363>121 for the quantifier and 363>97 for the qualifier.

The inter- and intra-assay CVs for serum corticosterone were 7.3 and 2.9%, respectively, and for cortisol concentrations were 13.6 and 9.6%, respectively on control level 1 (lowest concentration).

### Statistical analyses

All statistical analyses were conducted in R version 3.6.2 (26). Prior to the statistical analyses, the normality of the continuous variables was assessed using distribution graphs (QQ-plots, histograms, and box plots) as well as the Shapiro–Wilk test. None of the continuous variables shown in Table 1 were normally distributed and are thus presented as medians (25th–75th percentiles). Therefore, the Mann–Whitney *U-*test was used to assess the statistical differences in the continuous variables between men and women (Table 1). Differences in the categorical variables (prescribed medication, prevalence of metabolic syndrome, and lifestyle factors) are presented as observations/total non-missing observations (*n*/*N* (%)) and were compared between men and women using a chi-square test (Table 1).

**Table 1:** Characteristics of the study participants.

	*n*	All (*n* =1104)	Men (*n* =316)	Women (*n* =788)	*P*
Age and adiposity					
Age (years)	1096	51.9 (46.8–57.0)	53.0 (48.1–59.0)	51.0 (46.0–56.3)	**<0.001**
BMI (kg/m^2^)	1094	30.7 (25.2–35.8)	25.2 (21.0–30.1)	32.8 (28.1–37.3)	**<0.001**
Waist circumference (cm)	1066	95.0 (86.0–104.5)	93.0 (81.8–103.8)	96.0 (87.2–105.0)	**0.029**
Blood pressure					
Systolic blood pressure (mmHg)	1076	128.2 (115.5–142.8)	131.0 (118.2–145.0)	127.0 (114.0–142.5)	**0.017**
Diastolic blood pressure (mmHg)	1076	86.0 (78.0–94.7)	87.5 (80.5–96.0)	85.5 (76.5–94.0)	**0.003**
Glycaemia and insulin resistance					
Fasting glucose (mmol/L)	1038	5.0 (4.5–5.5)	5.0 (4.4–5.4)	5.0 (4.5–5.6)	0.147
Fasting insulin (µIU/mL)	964	8.0 (4.4–13.9)	5.8 (2.3–11.7)	9.1 (5.2–14.6)	**<0.001**
2-h glucose (mmol/L)	636	5.9 (4.7–7.1)	5.7 (4.3–6.7)	6.0 (5.0–7.3)	**<0.001**
HbA1c (%)	873	5.8 (5.4–6.2)	5.5 (5.2–6.1)	5.8 (5.5–6.3)	**<0.001**
HOMA2-IR	938	1.0 (0.6–1.8)	0.7 (0.3–1.5)	1.2 (0.7–1.9)	**<0.001**
Matsuda index (OGTT-derived insulin sensitivity)	555	5.7 (3.1–9.4)	6.7 (3.4–12.4)	4.8 (2.8–7.5)	**<0.001**
Serum lipids					
Total cholesterol (mmol/L)	1012	4.3 (3.7–5.0)	4.1 (3.5–4.8)	4.4 (3.8–5.1)	**<0.001**
LDL cholesterol (mmol/L)	1012	2.7 (2.1–3.3)	2.4 (1.8–3.0)	2.8 (2.2–3.4)	**<0.001**
HDL cholesterol (mmol/L)	1013	1.2 (1.0–1.4)	1.2 (1.0–1.5)	1.2 (1.0–1.4)	0.148
Triglycerides (mmol/L)	1012	0.9 (0.7–1.2)	0.9 (0.7–1.3)	0.9 (0.7–1.2)	0.253
Glucocorticoids					
Blood sampling time (min)	1027	90.0 (60.0–140.0)	54.0 (40.0–74.8)	113.0 (75.0–165.0)	**<0.001**
Corticosterone (nmol/L)	924	6.3 (3.5–13.6)	5.0 (3.3–9.0)	6.8 (3.6–17.9)	**<0.001**
Cortisol (nmol/L)	972	182.5 (79.5–342.5)	153.8 (76.3–295.8)	201.2 (82.5–371.9)	**0.002**
Medication					
Blood pressure med (*n*/*N* (%))	854	290/854 (34.0)	84/310 (27.1)	206/544 (37.9)	**0.002**
Diabetes med (*n*/*N* (%))	792	73/792 (9.2)	19/310 (6.1)	54/482 (11.2)	**0.022**
Cholesterol-lowering med (*n*/*N* (%))	895	61/895 (6.8)	21/310 (6.8)	40/585 (6.8)	1.000
Metabolic syndrome					
Prevalence (2009 harmonized definition) (*n*/*N* (%))	1066	440/1066 (40.7)	83/316 (26.3)	357/750 (47.6)	**<0.001**
Lifestyle					
Smoking (*n*/*N* (%))	1084	224/1084 (20.7)	160/315 (50.8)	64/769 (8.3)	**<0.001**
Alcohol (*n*/*N* (%))	674	329/674 (48.8)	226/315 (71.7)	103/359 (28.7)	**<0.001**

Continuous data presented as median (25th–75th percentiles) and categorical data presented as *n* (%). A Wilcoxon rank sum test was used to statistically compare the continuous variables, whilst a chi-square test was used to statistically compare the categorical variables. Bold indicates *P* < 0.05.

Med, medication; min, minutes from the earliest sampling time (07:00 h); *n*, number of observations; *N*, Total number of non-missing observations; *P*, *P* value of the Wilcoxon rank sum statistical or chi-square test.

Logistic or linear regression models were used to determine associations between serum glucocorticoid concentrations and metabolic syndrome and related cardiometabolic risk factors, respectively. Blood sampling time, age, sex, alcohol, and smoking were used as covariates in all regression models. Prescribed medications for blood pressure, diabetes, and lipids were also used as covariates in regression models that included measures of blood pressure, measures of glycaemia and insulin resistance, and lipid profile. The associations between circulating glucocorticoids and adiposity are often confounded by the increased metabolic clearance of glucocorticoids within the adipose and liver tissues in obesity (27). Hence, BMI was included as an additional covariate in all models (except where BMI, waist circumference, and metabolic syndrome were the outcomes). For the regression models, all continuous dependent variables were log-transformed to achieve normal distributions prior to inclusion in the models. The regression models were first conducted in the whole study sample comprising both men and women. In these models, sex interactions were also tested, and the sex interaction *P* values were recorded as 'Sex Int'. All models showing evidence of sex interactions (Sex Int: *P* < 0.05) were subsequently stratified by sex, and each regression model was repeated in men and women separately.

To test the validity of the linear regression models, the presence of a linear relationship between the serum glucocorticoid concentrations and the relevant outcome variable was first confirmed in each model using scatter plots. The absence of multicollinearity within each model was confirmed by assessing the variance inflation factor (VIF) values (all VIF < 3.0). Finally, the validity of each linear regression model was confirmed by testing the normality of the residuals.

The tested outcome variables included metabolic syndrome, BMI, waist circumference, systolic and diastolic blood pressure, fasting glucose and insulin, 2-h glucose, HOMA2-IR, Matsuda index, and serum lipids (triglyceride, total-, LDL- and HDL-cholesterol).

**Figure 2:**
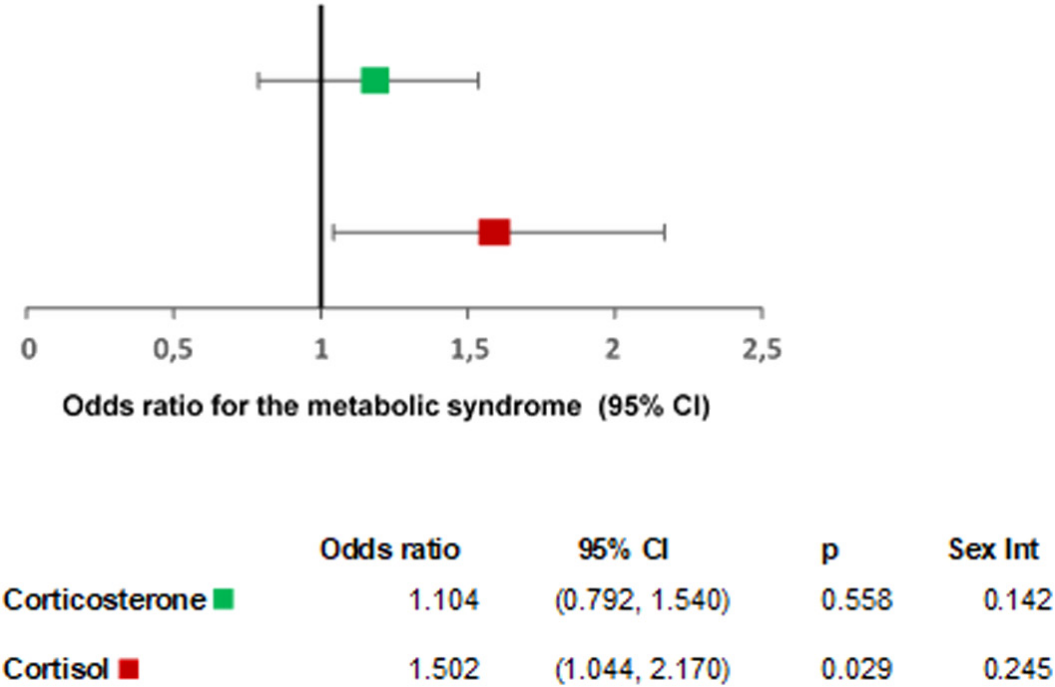
Associations between glucocorticoids and metabolic syndrome. *P*, *P* value for the logistic regression model; Sex Int, *P* value for sex interaction Models adjusted for age, blood sampling time, sex, smoking, and alcohol.

Data from the logistic regression models are presented as the odds ratio presenting with metabolic syndrome, for both corticosterone and cortisol, with adjustment for potential confounders (age, sex, blood sampling time, smoking, and alcohol) (Fig. 2). For the linear models, the beta coefficients and 95% CI for the various outcome measures listed above are presented in Table 2 adjusted for the potential confounders only (referred as Model 1) and for the potential confounders plus BMI (referred as Model 2).

**Table 2:** Associations of glucocorticoids with cardiometabolic risk factors in African men and women.

	Model 1 (adjusted for confounders)			Model 2 (adjusted for confounders and BMI)		
	Beta (95% CI)	*P*	Sex Int	Beta (95% CI)	*P*	Sex Int
BMI						
Corticosterone	−0.046 (−0.096, 0.004)	0.071	0.063			
Cortisol	−0.011 (−0.064, 0.041)	0.679	**0.040**			
Systolic BP						
Corticosterone	0.007 (−0.029, 0.043)	0.714	0.656	0.014 (−0.021, 0.049)	0.430	0.956
Cortisol	0.042 (0.005, 0.079)	**0.028**	0.955	0.043 (0.007, 0.080)	**0.018**	0.634
Diastolic BP						
Corticosterone	0.009 (−0.023, 0.041)	0.572	0.897	0.016 (−0.016, 0.047)	0.324	0.775
Cortisol	0.051 (0.017, 0.085)	**0.004**	0.918	0.052 (0.019, 0.085)	**0.002**	0.546
Fasting glucose						
Corticosterone	−0.008 (−0.055, 0.039)	0.739	**0.009**	−0.001 (−0.048, 0.046)	0.953	**0.008**
Cortisol	0.042 (−0.010, 0.094)	0.110	**<0.001**	0.044 (−0.008, 0.095)	0.097	**<0.001**
2-h glucose						
Corticosterone	−0.017 (−0.099, 0.065)	0.683	0.971	−0.001 (−0.082, 0.079)	0.972	0.824
Cortisol	−0.013 (−0.099, 0.074)	0.775	0.090	−0.010 (−0.095, 0.075)	0.810	**0.047**
HbA1c						
Corticosterone	−0.013 (−0.045, 0.019)	0.435	**0.003**	−0.010 (−0.042, 0.022)	0.543	**0.002**
Cortisol	−0.001 (−0.036, 0.035)	0.965	**0.003**	−0.001 (−0.036, 0.035)	0.974	**0.001**
Matsuda index						
Corticosterone	0.220 (0.034, 0.407)	**0.021**	0.444	0.134 (−0.036, 0.303)	0.122	0.921
Cortisol	0.083 (−0.117, 0.284)	0.415	0.572	0.048 (−0.133, 0.229)	0.603	0.848
LDL cholesterol						
Corticosterone	−0.095 (−0.193, 0.003)	0.057	**0.019**	−0.086 (−0.184, 0.012)	0.085	**0.029**
Cortisol	−0.138 (−0.241, −0.034)	**0.009**	0.565	−0.136 (−0.239, −0.034)	**0.009**	0.768
HDL cholesterol						
Corticosterone	0.018 (−0.066, 0.102)	0.678	0.263	−0.009 (−0.088, 0.071)	0.830	0.377
Cortisol	0.105 (0.017, 0.193)	**0.020**	**0.038**	0.102 (0.019, 0.186)	**0.017**	0.083

Beta, beta coefficient; Confounders, age, blood sampling time, sex, smoking, alcohol, and relevant medication; *P*:,*P* value for the linear regression model; Sex Int, *P* value for sex interaction. Bold indicates *P* < 0.05. .

## Results

### Participant characteristics

The characteristics of the whole sample, and men and women separately, are presented in Table 1, with a comparison of the categorized components of metabolic syndrome and related cardiometabolic risk factors presented in Supplementary Table 1 (see section on supplementary materials given at the end of this article). Women were younger than men (*P* < 0.001) and had a higher BMI (*P* < 0.001) and waist circumference (*P* = 0.029) (Table 1). Accordingly, a greater proportion of women was classified with obesity (65.2% vs 25.3%; *P* < 0.001) and with a waist circumference greater than that of metabolic syndrome cut points (88.3% vs 48.4%; *P* < 0.001) (Supplementary Table 1).

Despite differences in obesity, fasting glucose was similar between men and women. However, compared to men, women had higher fasting insulin, HOMA2-IR, 2-h glucose, HbA1c concentrations, and lower insulin sensitivity (Matsuda index) (all *P* < 0.001). Although a larger proportion of women was using diabetes medication (11.2% vs 6.1%; *P* = 0.022) than men (Table 1), when using metabolic syndrome criteria (fasting blood glucose ≥ 5.6 mmol/L and/or using diabetes medication) the proportion of men and women with elevated blood glucose did not differ (Supplementary Table 1).

Although HDL-cholesterol and triglycerides did not differ by sex, total cholesterol and LDL-cholesterol concentrations were higher in women compared to men (*P* < 0.001 for both) (Table 1). Moreover, a greater proportion of women had low HDL cholesterol (57.0% vs 18.0%), but the prevalence of elevated triglycerides was greater in men (15.2 vs 8.2%) (*P* < 0.001 for both) (Supplementary Table 1). There were no observed differences in the use of cholesterol-lowering medication between the sexes.

Systolic and diastolic blood pressure were both higher in men (*P* = 0.017 and *P* = 0.003, respectively) (Table 1), and this finding was accompanied by a greater proportion of men having elevated systolic (≥130 mmHg and/or using blood pressure medication, 53.0% vs 46.3%; *P* = 0.017), diastolic (≥85 mmHg and/or using blood pressure medication, 61.3 vs 52.8%; *P* = 0.003), and overall blood pressure (73.4% vs 66.5%; *P* = 0.030) (Supplementary Table 1). In contrast, a greater proportion of women used blood pressure medication (37.9% vs 27.1%; *P* = 0.002).

A greater proportion of women than men were classified as having metabolic syndrome (47.6% vs 26.3%; *P* < 0.001). Meanwhile, a greater proportion of men smoked (50.8% vs 8.3%; *P* < 0.001) and drank alcohol (71.7% vs 28.7%; *P* < 0.001) compared to women.

When comparing the serum glucocorticoid concentrations (Table 1), women had higher serum concentrations of both corticosterone (*P* < 0.001) and cortisol (*P* < 0.002). These differences were accompanied by later average blood sampling times (measured as minutes from the earliest blood sampling time) in women compared to men (*P* < 0.001). Notably, the sex differences in serum glucocorticoid concentrations were observed even after adjusting for sampling time, measures of adiposity (BMI and waist circumference), and/or other potential confounders (age, smoking, alcohol) (results not shown).

### Associations with metabolic syndrome and related cardiometabolic risk factors in black South Africans

Only regression models that either showed evidence of associations (*P* < 0.05) or sex-interaction (Sex Int: *P* <0.05) are presented in Fig. 2 and Table 2. The unadjusted models and all other models that were tested are presented in Supplementary Table 2. Figure 2 shows that serum cortisol but not corticosterone concentrations were associated with greater odds presenting with metabolic syndrome. The association between circulating glucocorticoids and metabolic syndrome did not differ by sex (Sex Int: *P* = 0.142 for corticosterone and *P* = 0.245 for cortisol).

**Figure 3:**
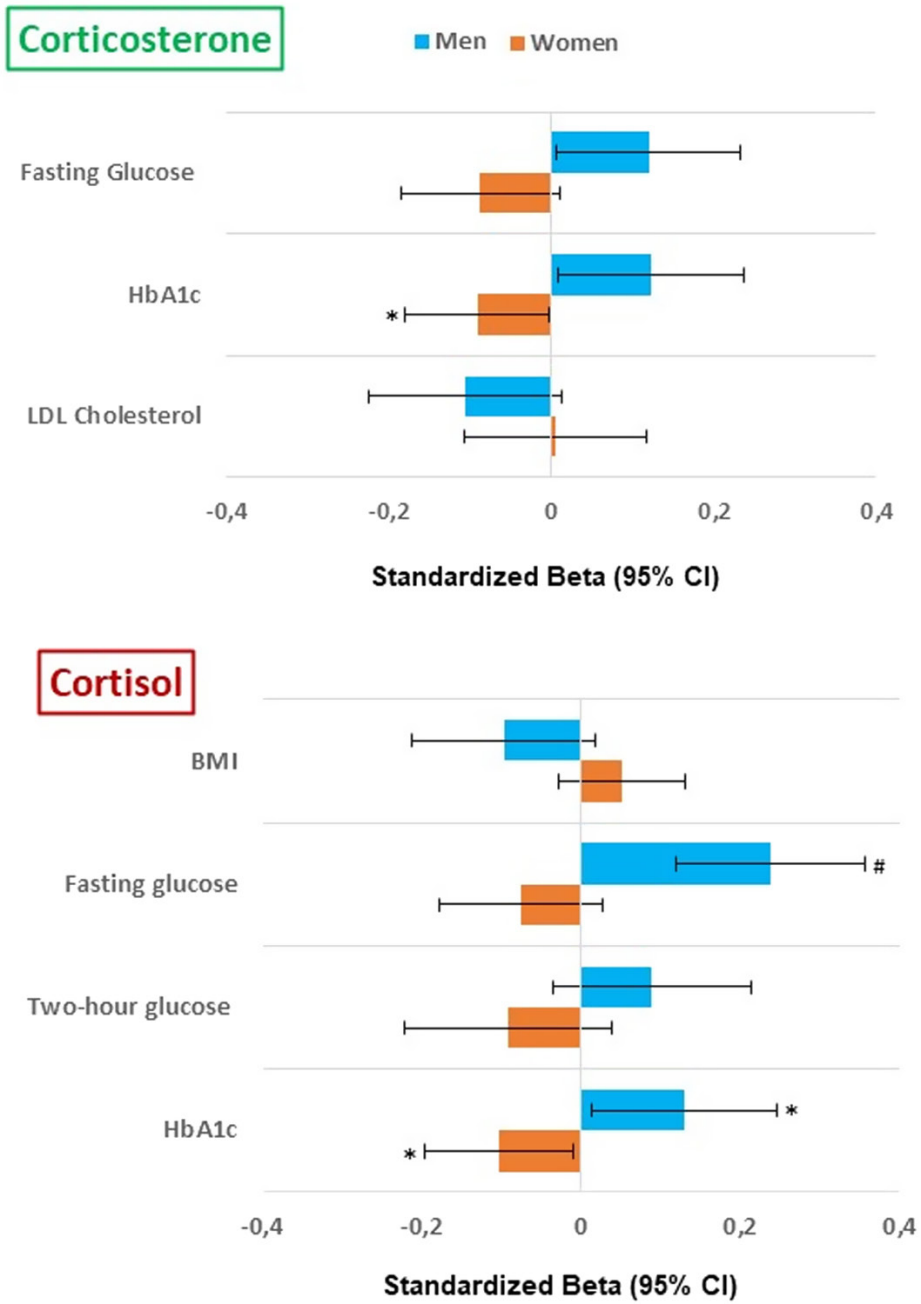
Sex-stratified associations of corticosterone and cortisol with cardiometabolic risk factors. Models adjusted for age, blood sampling time, smoking, alcohol, relevant medication, and BMI *P value < 0.05; #*P* value < 0.001.

There was no evidence of an association between corticosterone and cortisol and measures of adiposity (BMI and waist circumference) (Table 2). However, there was a sex-interaction for the association between serum cortisol concentration and BMI (Table 2), but the sex-stratified analyses revealed no sufficient evidence of association (Fig. 3). There was no evidence of sex interaction in the associations between either of the serum glucocorticoid measures and waist circumference.

Serum cortisol, but not corticosterone, concentrations were associated with higher systolic (*P* = 0.028) and diastolic (*P* = 0.004) blood pressure, even after adjusting for BMI (*P* = 0.018 and *P* = 0.002, respectively). These associations did not differ by sex (Table 2).

No evidence of association for serum corticosterone and cortisol concentrations was observed with measures of glycaemia in the combined sample of men and women. However, there was evidence of sex interactions for associations with fasting blood glucose (Sex Int: *P* = 0.009 and *P* < 0.001, respectively) and HbA1c (Sex Int: *P* = 0.003 for both) concentrations (Fig. 3 and Table 2). These sex interactions were present even after adjusting for BMI. Subsequent sex-stratified analyses revealed that both serum corticosterone and cortisol concentrations were associated with higher fasting blood glucose (*P* = 0.040 and *P* < 0.001, respectively) and HbA1c (*P* = 0.035 and *P* = 0.030, respectively) concentrations in men only, but lower HbA1c concentrations (*P* = 0.042 and *P* = 0.030, respectively) in women only (Fig. 3). Although the association between serum cortisol and 2-h OGTT glucose concentrations showed a sex-interaction after adjusting for BMI (Sex Int: *P* = 0.047), sex-stratified analyses revealed no evidence of association in either sex (Fig. 3 and Table 2).

Serum corticosterone, but not cortisol, was associated with higher insulin sensitivity (Matsuda index, *P* = 0.021), but the association was no longer present after adjusting for BMI (*P* = 0.122) (Table 2). Neither of the glucocorticoids were associated with fasting insulin concentrations or HOMA-IR. Moreover, there was no evidence of sex interactions for any measure of insulin resistance.

Serum cortisol concentrations were associated with lower LDL (*P* = 0.009) and higher HDL (*P* = 0.020) cholesterol (Table 2). These associations remained even after adjusting for BMI (*P* = 0.009 and 0.017, respectively). While serum corticosterone concentrations were not associated with any of the serum lipid concentrations, there was evidence of sex-interaction for LDL cholesterol (Sex Int: *P* = 0.019) which was not altered by the inclusion of BMI (Sex Int: *P* = 0.029) (Table 2). However, sex-stratified analyses revealed no evidence of sex-specific associations for LDL cholesterol concentrations (Fig. 3). In contrast, although there was evidence of a sex interaction for HDL cholesterol concentrations (Sex Int: *P* = 0.038), this was not observed after including BMI as a covariate.

## Discussion

We have shown for the first time that associations between circulating glucocorticoid concentrations and key cardiometabolic variables are both glucocorticoid- and sex-specific in black South Africans.

The higher prevalence of metabolic syndrome in women compared to men in the present study (45.8% vs 27.8%) was in accordance with previous studies in African populations (28). However, this is the first study to show that fasting serum cortisol concentrations were associated with greater odds of having metabolic syndrome in a combined sample of African men and women. This observation is in accordance with cross-sectional studies from Asian and European populations, which demonstrated that individuals with metabolic syndrome had higher circulating cortisol concentrations compared to those without metabolic syndrome (6, 29). The relationship between circulating cortisol concentrations and metabolic syndrome may have been attributed to underlying associations of cortisol with the individual components of metabolic syndrome. In this study, the relationship between cortisol and metabolic syndrome was largely driven by blood pressure as there was no evidence of association with waist circumference or triglycerides, while the association was positive with HDL cholesterol and showed sex-specificity for fasting glucose.

The finding that cortisol, but not corticosterone, concentrations were associated with higher systolic and diastolic blood pressure was in accordance with previous studies demonstrating that circulating cortisol concentrations were associated with both systolic and diastolic blood pressure in Africans, Asians, and Europeans (19, 20, 21, 30, 31). A study that has investigated circulating corticosterone in Europeans demonstrated that the associations between corticosterone concentrations and systolic blood pressure were weaker than those demonstrated for cortisol, and corticosterone was not associated with systolic blood pressure in other independent European cohorts (Mackenzie SD, Crawford AA, Ackermann D, Schraut KE, Hayward C, Bolton JL, Saunders C, Al-Dujaili E, Dick B, Escher G, Vogt B, Pruijm M, Ponte B, Wilson JF, Strachan MWJ, Price JF, Phillips DIW, MacKenzie SM, Davies E, Reynolds RM & Walker BR, unpublished observations). Glucocorticoids influence blood pressure via several mechanisms which are thought to occur through binding of the GR or mineralocorticoid receptor (MR) in the kidneys or vascular system (32). For example, in the kidneys, glucocorticoids impair nitric oxide-mediated renal vasodilation (33) possibly by suppressing nitric oxide synthase, but also influence salt and water retention, ultimately increasing blood pressure (34). There is, however, no strong evidence to suggest that corticosterone and cortisol bind differently to the GR or MR and that these mechanisms are directly involved in the different associations of the two glucocorticoids with blood pressure (35, 36). It is unlikely that the glucocorticoid-specific associations with blood pressure were due to lower circulating corticosterone concentrations compared to cortisol (6.3 vs 182.5 nmol/L), as there was evidence of an association between corticosterone and measures of insulin sensitivity, in the present study. While future studies are still required to confirm that corticosterone induces the GR or MR response at these low concentrations in humans, animal studies have suggested that these glucocorticoid-specific associations may be related to differential transmembrane transport by ABC transporters (10, 37, 38). This hypothesis originates from the fact that cortisol and corticosterone differ in their susceptibility to transmembrane transport by ABC transporters. For example, ABCB1 is an ABC transporter that is highly expressed in the CNS, and selectively exports cortisol but not corticosterone across the blood-brain barrier (38). Consequently, while corticosterone concentrations are approximately 5% of the concentration of cortisol in the blood, the concentrations are much higher (~30%) in the cerebrospinal fluid (39). Thus, corticosterone may contribute disproportionately to the negative feedback control of the HPA axis within the CNS. As both glucocorticoids similarly bind to the GR and MR to increase blood pressure (33), higher circulating concentrations of corticosterone are accompanied by a more robust HPA axis regulatory response, in comparison to cortisol, to inhibit overall excess glucocorticoid production, ultimately lowering insulin resistance and blood pressure (38).

Additionally, it has been suggested that the relationship between glucocorticoids and elevated blood pressure may be partly mediated by adiposity (40), which is often associated with the other related cardiometabolic risk factors (1). However, adjusting for either BMI or waist circumference did not alter the relationship with blood pressure in our study. Further, there was no evidence of associations between the glucocorticoids and measures of adiposity in this study. It is possible that the overall effects on BMI and waist circumference were confounded by sex differences in body fat distribution and consequent differences in glucocorticoid clearance, as men generally accumulate more VAT compared to women, and glucocorticoid clearance is higher in VAT compared to SAT (41). In this study, men had lower a prevalence of obesity and elevated waist circumference compared to women (25.3 vs 65.2% and 48.4 vs 88.3%, respectively), and there was evidence of sex interaction in the association between cortisol and BMI (*P* value for sex interaction = 0.040). Although sex-stratified analyses revealed that the association between cortisol and BMI was negative in men but positive in women, there was not sufficient evidence (*P* > 0.05) for these relationships in our study sample. Hence, further studies that include larger samples of men and women are required to explore these sex-specific interactions.

Although there was no association between cortisol and insulin resistance, we have shown for the first time that corticosterone was associated with higher insulin sensitivity, as assessed using the Matsuda index. However, this relationship was not present after adjusting for BMI. This finding highlights the importance of including BMI as an additional covariate in glucocorticoid studies and supports the notion that circulating glucocorticoids are associated with adiposity but the relationship is confounded by other factors such as increased metabolic clearance in obesity and type 2 diabetes (27).

This study has also demonstrated that cortisol was associated with a favourable lipid profile (lower serum LDL and higher HDL cholesterol) in black South Africans. Studies from non-Africans that investigated the relationship between circulating cortisol and serum lipid concentrations are inconsistent (29, 30, 31, 42, 43). Although some studies reported no association between circulating cortisol and HDL cholesterol (29, 30), other studies reported an inverse association (31), whilst another reported a positive association (42). Likewise, while some studies have demonstrated a positive association between circulating cortisol and LDL cholesterol (43), other studies showed no evidence of this association (29, 30). These contradictory findings were thought to be related to differences in age and BMI between the various studies. However, in the present study, the associations between serum cortisol and HDL and LDL cholesterol were independent of BMI, negating the involvement of adiposity-related mechanisms. Indeed, further studies are necessary to understand the exact mechanisms involved.

In addition to glucocorticoid-specific associations, findings from the present study suggested for the first time that both serum corticosterone and cortisol showed sex-specific associations with measures of glycaemia. Both fasting serum cortisol and corticosterone concentrations were associated with higher fasting plasma glucose and HbA1c concentrations in men only, but lower HbA1c concentrations in women only. As these sex-specific associations were similar for both corticosterone and cortisol, mechanisms may involve sex differences in glucocorticoid signalling in the liver, as demonstrated in rodent models. Mice treated with dexamethasone (a synthetic glucocorticoid) demonstrated several sex-specific transcriptional responses in the hepatic tissue (44, 45). Moreover, another study showed that chronic exposure to excess glucocorticoid concentrations increased circulating glucose concentrations in male mice, but female mice were protected from these effects (46). Both corticosterone and cortisol are known to increase circulating glucose concentrations by promoting gluconeogenesis in the liver (47). Hence, sex disparities in glucocorticoid signalling within the liver may partly explain the observed sex-specific associations between circulating glucocorticoids and measures of glycaemia, which in turn may be mediated by sex hormones and body fat distribution (48). Indeed, disparities in sex hormone and body fat distribution profiles are profound between men and women. In men, testosterone predominates, and fat is preferentially stored as VAT, whilst in women, oestrogen predominates, and fat is preferentially stored as SAT (45). Such differences may have explained the observed sex-specific associations in the present study. Both sex hormones and fat distribution have been shown to potentially modulate responses to glucocorticoids (48). For example, oestrogen increases corticosteroid-binding globulin, the primary glucocorticoid-binding protein in circulation, consequently increasing biologically active glucocorticoids (49). Likewise, the GR is more highly expressed in VAT than SAT (50). Therefore, further studies are required to investigate the role of hepatic glucocorticoid signalling and interactions with sex hormones and body fat distribution, in the observed sex-specific associations.

This study has some limitations. The role of body distribution, menopausal status, and oral contraceptive use was not assessed in the present study. These factors are known to influence circulating glucocorticoid concentrations and may have influenced some of the reported results (41, 51). Women were overrepresented in the present study, and this is common in many epidemiological studies in Africans (52). Although associations with several outcome variables were tested, adjustment for multiple testing was not conducted. However, as the outcomes are all thought to be interrelated cardiometabolic risk factors, adjusting for multiple testing may be too conservative and limit the probability of showing true effects. Additionally, circulating glucocorticoids are often measured at a set time (e.g. 08:00 or 09:00 h) due to their diurnal nature. Notably, blood sample times in the present study varied widely. However, all samples were taken in the morning and blood sampling time was adjusted for in all models.

Regardless of these limitations, this is the largest study to date that has compared the associations of both corticosterone and cortisol concentrations with metabolic syndrome and its components and related cardiometabolic risk factors in African men and women. This study used a UPLC-MS method for measuring glucocorticoid concentrations which is preferred due to its high specificity and sensitivity. In addition, all known confounders including sampling time, age, sex, relevant medication, smoking, alcohol, and adiposity, were adjusted for in the statistical analyses.

In conclusion, this study shows that circulating glucocorticoid concentrations are associated with the metabolic syndrome and related cardiometabolic variables in African men and women, but that these associations were glucocorticoid- and sex-specific. Future studies should investigate the mechanisms involved, and ensure that both cortisol and corticosterone, and sex interactions are explored.

## Supplementary Material

Supplementary Table 1 Summary of the categorical variables of the study sample.

Supplementary Table 2 All regression models tested in the combined sample of African men and women.

## Declaration of interest

The authors declare that there is no conflict of interest that could be perceived as prejudicing the impartiality of the research reported.

## Funding

The study was funded by the Academy of Medical Sciences http://dx.doi.org/10.13039/501100000691 Newton Advanced Fellowship and the South African National Research Foundation http://dx.doi.org/10.13039/501100001321 (NRF). We also acknowledge the SAMRC, with funds received from the South African National Department of Health http://dx.doi.org/10.13039/100009041, the UKMRC, the Newton Fund http://dx.doi.org/10.13039/100010897, GSK and NRF. A A C and B R W were supported by grants from British Heart Foundationhttp://dx.doi.org/10.13039/501100000274 and Wellcome Trust http://dx.doi.org/10.13039/100010269. Opinions expressed and conclusions arrived at are those of the author and are not necessarily attributed to the research funders.

## Author contribution statement

S N D, J H G, Z L, A A C, and B R W were responsible for the study conception and planning. L K M, N J C, and S A N were involved in sample and data collection. S N D and T S were responsible for measuring glucocorticoids. S N D, J H G, and Z L were involved in the data analyses. All authors were involved in the interpretation of the results and the writing of the manuscript.
