# Supplementary material for: Glucocorticoids associate with cardiometabolic risk factors in black South Africans
Source: Endocr Connect. 2021 Jul 14;10(8):873–84. doi: 10.1530/EC-21-0195 (PMC8346194; doi:10.1530/EC-21-0195)
Supplement: Supplementary Table 1 Summary of the categorical variables of the study sample. [file supplementary_table_1.pdf]

Supplementary Table 1 Summary of the categorical variables of the study sample.

	n	All (n =1104)	Men (n =316)	Women (n =788)	p
**<u>Adiposity</u>**					
**BMI categories**	1094				**<0.001**
Underweight (< 18.5 kg/m^2)		42 (3.8)	31 (9.8)	11 (1.4)	
Normal (18.5 – 24.9 kg/m^2)		222 (20.3)	125 (39.5)	97 (12.5)	
Overweight (25–29.9 kg/m^2)		243 (22.2)	80 (25.3)	163 (21.0)	
Obese (≥30 kg/m^2)		587 (53.7)	80 (25.3)	507 (65.2)	
**Elevated waist circumference** (≥80 cm in women; ≥94 cm in men)	1066	849 (79.6)	153 (48.4)	696 (88.3)	**<0.001**
**<u>Blood pressure (BP)</u>**					
**Elevated systolic BP** (≥130 mmHg)	1076	519 (48.2)	167 (53.0)	352 (46.3)	**0.017**
**Elevated diastolic BP** (≥85 mmHg)	1076	595 (55.3)	193 (61.3)	402 (52.8)	**0.003**
**Elevated BP** (Elevated systolic and/or diastolic BP and/or using of BP medication)	1104	756 (68.5)	232 (73.4)	524 (66.5)	**0.030**
**<u>Glycaemia</u>**					
**Fasting glucose (FG) categories**	1038				**0.023**
Normal FG (<5.6 mmol/L)		791 (76.4)	251 (79.4)	540 (74.7)	
Impaired FG (5.6-6.9 mmol/L)		161 (15.5)	50 (15.8)	111 (15.4)	
Elevated FG (≥7.0 mmol/L)		86 (8.2)	15 (4.7)	71 (9.8)	
**Elevated blood glucose** (Elevated FG and/or using diabetes medication)	1104	269 (24.4)	74 (23.4)	195 (24.7)	**0.699**
**<u>Serum lipids</u>**					
**Elevated fasting triglycerides** (≥1.7 mmol/L)	1012	105 (10.4)	48 (15.2)	57 (8.2)	**<0.001**
**Reduced fasting HDL-cholesterol** (<1.0 mmol/L in men, <1.3 mmol/L in women)	1013	455 (44.9)	57 (18.0)	398 (57.0)	**<0.001**

Data presented as n (%). All categorical variables were statistically compared between sexes using a chi-square test. **N:** Number of observations; **BMI:** Body Mass Index; blood pressure; **BP:** Blood pressure; **FG:** Fasting glucose; **HDL:** High Density Lipoprotein; **P**: P value of the chi-square test conducted.
