# Supplementary material for: Glucocorticoids associate with cardiometabolic risk factors in black South Africans
Source: Endocr Connect. 2021 Jul 14;10(8):873–84. doi: 10.1530/EC-21-0195 (PMC8346194; doi:10.1530/EC-21-0195)
Supplement: Supplementary Table 2 All regression models tested in the combined sample of African men and women. [file supplementary_table_2.pdf]

**Supplementary Table 2** All regression models tested in the combined sample of African men and women.

| | | **Unadjusted Model** | | | | **Model 1 (Adjusted for confounders)** | | | | **Model 2 (Adjusted for confounders and BMI)** | | | |
|---|---|---|---|---|---|---|---|---|---|---|---|---|---|
| | **Dependent variable** | **Beta/OR** | **95% CI** | **p** | **Sex Int** | **Beta/OR** | **95% CI** | **p** | **Sex Int** | **Beta/OR** | **95% CI** | **p** | **Sex Int** |
| **Corticosterone** | BMI | 0.005 | (-0.033, 0.043) | 0.794 | 0.173 | -0.046 | (-0.096, 0.004) | 0.071 | 0.063 | N/A | N/A | N/A | N/A |
| | Waist circumference | -0.011 | (-0.034, 0.013) | 0.375 | 0.274 | -0.030 | (-0.065, 0.005) | 0.089 | 0.123 | N/A | N/A | N/A | N/A |
| | Systolic BP | 0.010 | (-0.015, 0.035) | 0.437 | 0.633 | 0.007 | (-0.029, 0.043) | 0.714 | 0.656 | 0.014 | (-0.021, 0.049) | 0.430 | 0.956 |
| | Diastolic BP | -0.003 | (-0.025, 0.020) | 0.809 | 0.786 | 0.009 | (-0.023, 0.041) | 0.572 | 0.897 | 0.016 | (-0.016, 0.047) | 0.324 | 0.775 |
| | Fasting glucose* | -0.032 | (-0.071, 0.007) | 0.113 | **0.031** | -0.008 | (-0.055, 0.039) | 0.739 | **0.009** | -0.001 | (-0.048, 0.046) | 0.953 | **0.008** |
| | Fasting insulin | 0.092 | (-0.040, 0.223) | 0.172 | 0.064 | -0.119 | (-0.301, 0.063) | 0.200 | 0.219 | -0.031 | (-0.191, 0.129) | 0.705 | 0.446 |
| | Two-hour glucose | -0.008 | (-0.093, 0.077) | 0.854 | 0.791 | -0.017 | (-0.099, 0.065) | 0.683 | 0.971 | -0.001 | (-0.082, 0.079) | 0.972 | 0.824 |
| | HbA1c* | -0.016 | (-0.047, 0.014) | 0.301 | **0.007** | -0.013 | (-0.045, 0.019) | 0.435 | **0.003** | -0.010 | (-0.042, 0.022) | 0.543 | **0.002** |
| | HOMA2-IR | 0.077 | (-0.057, 0.211) | 0.261 | 0.083 | -0.129 | (-0.312, 0.055) | 0.170 | 0.300 | -0.045 | (-0.205, 0.116) | 0.586 | 0.551 |
| | Matsuda index | 0.228 | (0.042, 0.415) | **0.017** | 0.560 | 0.220 | (0.034, 0.407) | **0.021** | 0.444 | 0.134 | (-0.036, 0.303) | 0.122 | 0.921 |
| | Total cholesterol | -0.009 | (-0.047, 0.029) | 0.653 | 0.077 | -0.053 | (-0.108, 0.001) | 0.056 | 0.060 | -0.053 | (-0.108, 0.002) | 0.060 | 0.077 |
| | LDL cholesterol* | -0.007 | (-0.075, 0.062) | 0.850 | **0.012** | -0.095 | (-0.193, 0.003) | 0.057 | **0.019** | -0.086 | (-0.184, 0.012) | 0.085 | **0.029** |
| | HDL cholesterol | -0.024 | (-0.084, 0.036) | 0.431 | **0.042** | 0.018 | (-0.066, 0.102) | 0.678 | 0.263 | -0.009 | (-0.088, 0.071) | 0.830 | 0.377 |
| | Triglycerides | 0.060 | (-0.014, 0.135) | 0.110 | **0.013** | -0.066 | (-0.177, 0.046) | 0.247 | 0.063 | -0.044 | (-0.153, 0.066) | 0.431 | 0.116 |
| | Metabolic syndrome | 0.983 | (0.730, 1.321) | 0.908 | 0.392 | 1.104 | (0.792, 1.540) | 0.558 | 0.142 | NA | NA | NA | NA |
| **Cortisol** | BMI* | 0.017 | (-0.024, 0.059) | 0.414 | 0.086 | -0.011 | (-0.064, 0.041) | 0.679 | **0.040** | N/A | N/A | N/A | N/A |
| | Waist circumference | 0.008 | (-0.018, 0.034) | 0.540 | 0.085 | 0.000 | (-0.016, 0.016) | 0.984 | 0.055 | N/A | N/A | N/A | N/A |
| | Systolic BP | 0.036 | (0.009, 0.063) | **0.009** | 0.942 | 0.042 | (0.005, 0.079) | **0.028** | 0.955 | 0.043 | (0.007, 0.080) | **0.018** | 0.634 |
| | Diastolic BP | 0.046 | (0.022, 0.070) | **$1.790 \times 10^{-4}$** | 0.954 | 0.051 | (0.017, 0.085) | **0.004** | 0.918 | 0.052 | (0.019, 0.085) | **0.002** | 0.546 |
| | Fasting glucose* | 0.027 | (-0.017, 0.072) | 0.229 | **0.010** | 0.042 | (-0.010, 0.094) | 0.110 | **<0.001** | 0.044 | (-0.008, 0.095) | 0.097 | **<0.001** |
| | Fasting insulin | 0.040 | (-0.106, 0.186) | 0.589 | **0.027** | -0.065 | (-0.261, 0.131) | 0.516 | 0.073 | -0.055 | (-0.226, 0.117) | 0.534 | 0.352 |
| | Two-hour glucose* | 0.032 | (-0.054, 0.118) | 0.467 | 0.255 | -0.013 | (-0.099, 0.074) | 0.775 | 0.090 | -0.010 | (-0.095, 0.075) | 0.810 | **0.047** |
| | HbA1c* | -0.009 | (-0.044, 0.026) | 0.619 | **0.022** | -0.001 | (-0.036, 0.035) | 0.965 | **0.003** | -0.001 | (-0.036, 0.035) | 0.974 | **0.001** |

| | | | | | | | | | | | | | |
|---|---|---|---|---|---|---|---|---|---|---|---|---|---|
| HOMA2-IR | 0.025 | (-0.124, 0.175) | 0.739 | 0.072 | -0.057 | (-0.254, 0.141) | 0.573 | 0.241 | -0.036 | (-0.208, 0.137) | 0.685 | 0.658 |
| Matsuda index | -0.054 | (-0.248, 0.141) | 0.589 | 0.128 | 0.083 | (-0.117, 0.284) | 0.415 | 0.572 | 0.048 | (-0.133, 0.229) | 0.603 | 0.848 |
| Total cholesterol | 0.007 | (-0.037, 0.050) | 0.764 | 0.955 | -0.048 | (-0.106, 0.010) | 0.102 | 0.690 | -0.048 | (-0.106, 0.010) | 0.104 | 0.573 |
| LDL cholesterol | -0.007 | (-0.082, 0.067) | 0.846 | **0.038** | -0.138 | (-0.241, -0.034) | **0.009** | 0.565 | -0.136 | (-0.239, -0.034) | **0.009** | 0.768 |
| HDL cholesterol | 0.037 | (-0.027, 0.102) | 0.254 | **0.014** | 0.105 | (0.017, 0.193) | **0.020** | **0.038** | 0.102 | (0.019, 0.186) | **0.017** | 0.083 |
| Triglycerides | 0.073 | (0.071, -0.006) | 0.152 | 0.651 | -0.032 | (-0.146, 0.081) | 0.577 | 0.708 | -0.030 | (-0.141, 0.081) | 0.598 | 0.394 |
| Metabolic syndrome | 1.397 | (1.005, 1.946) | 0.047 | 0.251 | 1.502 | (1.044, 2.170) | 0.029 | 0.245 | NA | NA | NA | NA |

**Unadjusted Model:** Unadjusted linear/logistic regression model; **Model 1 (Adjusted for confounders):** Linear/logistic regression model adjusted for age, sex, time of blood sample (calculated as minutes from the earliest sample (07h00)), smoking, alcohol, and relevant chronic medication; **Model 2 (Adjusted for confounders and BMI):** Linear/logistic regression model adjusted the above confounders and BMI. **Beta/OR:** regression co-efficient/odds ratio value of the linear/logistic model; **95% CI:** 95% confidence intervals; **p:** P value of the regression model**;** **Sex Int:** P value for the sex interaction.
